# Development and Characterization of EST-SSR Markers From RNA-Seq Data in *Phyllostachys violascens*

**DOI:** 10.3389/fpls.2019.00050

**Published:** 2019-02-01

**Authors:** Kai Cai, Longfei Zhu, Keke Zhang, Ling Li, Zhongyu Zhao, Wei Zeng, Xinchun Lin

**Affiliations:** ^1^Sino-Australia Plant Cell Wall Research Centre, State Key Laboratory of Subtropical Silviculture, Zhejiang A & F University, Lin’an, China; ^2^Zhejiang Provincial Collaborative Innovation Center for Bamboo Resources and High-Efficiency Utilization, Zhejiang A & F University, Lin’an, China; ^3^Department of Genome Biology, Adam Mickiewicz University, Poznań, Poland

**Keywords:** *Phyllostachys violascens*, transcriptome, microsatellites, varieties, genetic diversity

## Abstract

Bamboo are woody grass species containing important economic and ecological values. Lei bamboo (*Phyllostachys violascens*) is a kind of shoot-producing bamboo species with the highest economic yield per unit area. However, identifying different varieties of Lei bamboo based on morphological characteristics is difficult. Microsatellites play an important role in plant identification and genetic diversity analysis and are superior to other molecular markers. In this study, we identified 18,356 expressed sequence tag-simple sequence repeat (EST-SSR) loci in Lei bamboo transcriptome data. A total of 11,264 primer pairs were successfully designed from unigenes of all EST-SSR loci, and 96 primer pairs were randomly selected and synthesized. A total of 54 primer pairs were used for classifying 16 Lei bamboo varieties and 10 different *Phyllostachys* species. The number of polymorphism alleles among the 54 primer pairs ranged from 3 to 12 for *P. violascens* varieties and 3 to 20 for *Phyllostachys*. The phylogenetic tree based on polymorphism alleles successfully distinguished 16 *P. violascens* varieties and 10 *Phyllostachys* species. Our study provides abundant EST-SSR resources that are useful for genetic diversity analysis and molecular verification of bamboo and suggests that SSR markers developed from Lei bamboo are more efficient and reliable than ISSR, SRAP or AFLP markers.

## Introduction

Bamboo is an economically important member of the woody grasses. It includes 88 genera and more than 1,400 species worldwide; 34 genera and 534 species are in China (Wu and Raven, 2006). Given the considerably fast growth, strong carbon fixation capability and edible shoots, bamboo has worldwide ecological and economic value.

The classification and nomenclature of plants are mainly based on morphological characteristics, such as roots, stems, leaves and flowers (Lichtenthaler, 1987). Flower morphology is the most important (PDCress and Staden, 1998). The identification and classification of bamboo is vital for germplasm collection and conservation (Soderstrom and Calderon, 1979). However, bamboo has a long juvenile phase, with the flowering interval of some species being up to 120 years (Janzen, 1976), which prevents the classification of bamboo when using only flower morphology. Moreover, morphological characteristics sometimes are not very reliable because they are affected by ecological factors, which leads to confused classification and reclassification of various accessions of bamboo (Das et al., 2007).

Microsatellites or simple sequence repeats (SSRs), characterized by high polymorphism, wide distribution in genomes, co-dominance and reproducibility, have been frequently used for genetic analysis (Goldstein and Schlötterer, 1999). For instance, 69 varieties of taro (*Colocasia esculenta*) were successfully separated by expressed sequence tag-SSR (EST-SSR) primers (You et al., 2015). Eleven varieties of *Lycium* were verified by EST-SSRs (Chen et al., 2017). SSR markers have been used to study genetic diversity, genetic distance and classification of bamboo species (Kaneko et al., 2008; Mukherjee et al., 2010; Lin et al., 2011; Jiang et al., 2013; Dong and Yang, 2014; Zhao et al., 2015; Abhishek et al., 2016).

Lei bamboo (*Phyllostachys violascens*) has higher economic value per unit area than other bamboo species with edible shoots (Liu et al., 2001). Lei bamboo has many varieties that differ in shoot time, shoot yield per unit area and pathogen resistance. Lin et al. (2011) classified different *P. violascens* varieties by using inter-simple sequence repeat (ISSR), sequence-related amplified polymorphism (SRAP), and amplified fragment length polymorphism (AFLP) markers. However, some varieties cannot be differentiated by just one molecular marker method (Lin et al., 2011). Therefore, the development of accurate and efficient molecular markers for classifying different varieties in Lei bamboo is important.

Previously, we sequenced the transcriptome of *P. violascens* (GenBank Accession No. SRX5137626) by using Illumina Truseq and assembled 132,678 unigenes. We identified EST-SSR loci as well as primer pairs based on these data. The major objective of this study was to develop efficient EST-SSR markers for classifying Lei bamboo varieties as well as *Phyllostachys* species. These markers may be applicable for bamboo taxonomic study, genetic diversity analysis and evolutionary research.

## Materials and Methods

### Plant Materials

Young leaves of 16 varieties of *P. violascens* (cv. Jianye, cv. Panggan, cv. Violascens, cv. Notata, cv. Zaoyuanzhu, cv. Flavistriatus, cv. Hongke, cv. Xiye, cv. Viridisulcata, cv. Linanensis, cv. Kuoyeqingtou, cv. Anhuihongke, cv. Flavicaginis, cv. Qingke, cv. Atrovaginis, cv. Anhuiensis) were sampled from the Bamboo Garden of Zhejiang Agriculture and Forestry University (Lin et al., 2011). Young leaf samples of nine *Phyllostachys* species (*P. glabrata, P. verrucosa, P. bambusoides, P. aurea, P. edulis, P. virella, P. rivalis, P. parvifolia*, and *P. nidularia*) were collected from the China Bamboo Expo Park in Huzhou, Zhejiang province, China.

### Marker Loci Detection and SSR Primer Pair Design

MicroSAtellite (MISA)^1^ was used for SSR mining in the assembled contigs (Thiel et al., 2003). The minimum number of repeats used to select the SSRs was 10 for mononucleotide repeats, 6 for dinucleotide repeats, and 5 for tri-, tetra-, penta-, and hexanucleotide repeats. The Perl program Primer3.0^2^ (Rozen and Skaletsky, 2000) was used to design Primer pairs with the design principles of length 18 to 27 bp and about 20 bp, melting temperature (Tm) 57°C to 63°C, and PCR product size 100 to 280 bp.

### DNA Extraction and EST-SSR Marker Amplification

Total genomic DNA was extracted by using the cetyltrimethyl ammoniumbromide method (Porebski et al., 1997) and verified by electrophoresis on 1% agarose gel. PCR amplification was carried out in a 20-μl volume containing 50 ng template DNA, 2 μl of 10 × PCR buffer (Mg^2+^ free), 1.2 μl MgCl_2_ (20 mM), 1 μl sense primer (10 pmol), 1 μl anti-sense primer (10 pmol), 2 μl dNTP (10 mM), and 0.5 μl rTaq DNA polymerase (5 U, TAKARA). The PCR program consisted of an initial step of 95°C for 5 min, followed by 33 cycles of 95°C for 30 s, 55°C for 30 s, and 72°C for 1 min and a final extension at 72°C for 10 min (Rossetto, 2001). The PCR products were loaded on 1-mm-thick non-denaturing gels of 8% polyacrylamide (Acr/Bis = 29:1). The electrophoresis buffer contained 1 × TBE (100 mM Tris–HCl, 83 mM boric acid, 1 mM Na_2_EDTA, pH 8.0) (Han et al., 2010); the 20 bp DNA Ladder Dye Plus (Takara Biomedical Technology, Beijing) was used as a size standard.

### PCR Product Sequencing

Silver nitrate stain gel was used for selecting suitable SSR primers (Panaud et al., 1996). The desired bands were accurately excised and subsequently purified by using the SanPrep Column DNA Gel Extraction Kit (Sangon, Shanghai) (Tang et al., 2010). The purified DNA bands were ligated with pMD-18-T vector (TaKaRa, Beijing) and sequenced by the Sanger method (Tang et al., 2010; Zhai et al., 2014). The PCR sequence similarity rate was more than 98% with transcriptome sequences applied.

### Data Processing and Genetic Analysis

According to PAGE results, the absence or presence of bands was scored as zero or one in all SSR loci and two binary qualitative data matrices were generated (Rohlf, 2000; You et al., 2015). Dendrograms were constructed on the basis of these two binary qualitative matrices with the method unweight pair group method with arithmetic mean (UPGMA) selected for clustering and maximum number of tied trees set to 100. Before dendrogram construction, pairwise coefficients were calculated according to the Jaccard similarity coefficient method (Kosman and Leonard, 2005). The above-mentioned data processing, coupled with principal coordinates analysis (PCoA) involved using NTSYSpc v2.1 (Rohlf, 2000). The bootstrapping analysis repeat was 1,000 performed with the software FREETREE V.0.9.1.50 (Pavlícek et al., 1999). Bootstrap values more than 50 are listed on the dendrogram (Zhu et al., 2012; Zhai et al., 2013). In this study, we constructed three phylogenetic trees: 16 *P. violascens* varieties independently, 10 *Phyllostachys* species independently and combination with 16 *P. violascens* varieties and other 9 *Phyllostachys* species.

## Results

### Distribution and Frequency of SSR Markers

A total of 132,678 transcriptome sequences were examined by MISA software, and 18,356 EST-SSRs loci were identified in *P. violascens*. Among these SSRs, 2,239 (11.76%) unigenes contained more than one SSR locus, and 903 SSRs were presented in compound formation (Appendix 1 and Appendix 2). These SSRs were further divided into six different types based on unit size, that is, 9,099 mononucleotide repeats (mono-), 4,449 dinucleotide repeats (di-), 4,521 trinucleotide repeats (tri-), 235 tetranucleotide repeats (tetra-), 39 pentanucleotide repeats (penta-) and 13 hexanucleotide repeats (hexa-), accounting for 49.57, 24.24, 24.63, 1.28, 0.21, and 0.07% of total SSRs, respectively (Appendix 1).

The repeat motif length of SSR ranged from 10 to 60 bp. The most abundant was 10 bp (4,462, 24.31%), followed by 15 bp (3,362, 18.32%), 12 bp (2,166,13.0%), and 18 bp (1,878, 10.23%). The 60 bp length was the longest SSR loci (Appendix 1).

Within these SSRs, 95 motif types were detected (Appendix 1). The frequency distribution of the 20 most abundant SSR classical repeat motifs is in Table 1. Among those motif types, A/T was the most abundant (43.73%), followed by AG/CT (15.93%) and C/G (5.84%).

**Table 1:** Frequency distribution of the 20 most frequent EST-SSR repeat motifs in *Phyllostachys violascens.*

	Repeats	Number of repeats of the motif																						
No.	motif	5	6	7	8	9	10	11	12	13	14	15	16	17	18	19	20	21	22	23	24	46	Total	Frequency
1	A/T	–	–	–	–	–	4314	1499	767	395	277	160	97	81	96	110	122	61	33	13	2		8027	43.73%
2	AG/CT	–	877	551	531	557	343	62	3	–	–	–	–	–	–	–	–	–	–	–	–	1	2925	15.93%
3	CCG/CGG	1177	376	91	3	–	–	–	–	–	–	–	–	–	–	–	–	–	–	–	–	–	1647	8.97%
4	C/G	–	–	–	–	–	148	135	99	84	77	71	61	72	77	101	71	52	15	7	2		1072	5.84%
5	AC/GT	–	360	188	140	102	105	33	1	–	–	–	–	–	–	–	–	–	–	–	–	–	929	5.06%
6	AGG/CCT	502	182	60	3	–	–	–	–	–	–	–	–	–	–	–	–	–	–	–	–	–	747	4.07%
7	AGC/CTG	441	136	53	3	–	–	–	–	–	–	–	–	–	–	–	–	–	–	–	–	–	633	3.45%
8	AAG/CTT	230	87	50	3	–	–	–	1	–	–	–	–	–	–	–	–	–	–	–	–	–	371	2.02%
9	AT/AT	–	199	77	28	20	17	19	–	–	–	–	–	–	–	–	–	–	–	–	–	–	360	1.96%
10	ACC/GGT	221	73	28	1	–	–	–	–	–	–	–	–	–	–	–	–	–	–	–	–	–	323	1.76%
11	AAC/GTT	187	58	13	2	–	–	–	–	–	–	–	–	–	–	–	–	–	–	–	–	–	260	1.42%
12	CG/CG	–	171	50	10	3	1	–	–	–	–	–	–	–	–	–	–	–	–	–	–	–	235	1.28%
13	ACG/CGT	160	44	18	–	–	–	–	–	–	–	–	–	–	–	–	–	–	–	–	–	–	222	1.21%
14	ATC/ATG	97	24	8	2	–	–	–	–	–	–	–	–	–	–	–	–	–	–	–	–	–	131	0.71%
15	AAT/ATT	61	30	17	2	–	–	–	–	–	–	1	–	–	–	–	–	–	–	–	–	–	111	0.60%
16	ACT/AGT	55	13	7	1	–	–	–	–	–	–	–	–	–	–	–	–	–	–	–	–	–	76	0.41%
17	AAAG/CTTT	33	1	–	–	–	–	–	–	–	–	–	–	–	–	–	–	–	–	–	–	–	34	0.19%
18	ATCC/ATGG	25	1	–	–	–	–	–	–	–	–	–	–	–	–	–	–	–	–	–	–	–	26	0.14%
19	AGGG/CCCT	19	3	–	–	–	–	–	–	–	–	–	–	–	–	–	–	–	–	–	–	–	22	0.12%
20	AAGG/CCTT	12	3	–	–	–	–	–	–	–	–	–	–	–	–	–	–	–	–	–	–	–	15	0.08%
	Other	155	27	1	1	2	2	2	–	–	–	–	–	–	–	–	–	–	–	–	–	–	190	1.04%
	Total	3375	2665	1212	730	684	616	251	104	84	77	72	61	72	77	101	71	52	15	7	2	1	18356	100.00%


A total of 11,264 primer pairs were successfully designed by using Primer 3.0 and two Perl scripts of p3_in.pl and p3_out.pl^3^ from 15,600 unigenes. However, the software primer modeling failed for 6,189 unigene sequences (Appendix 2). We randomly selected 96 SSR primer pairs from all SSR primer pairs. Finally, 54 optimal primer pairs (Appendix 3) were used to study a series of filters: (1) unsatisfactory amplification, (2) unspecific amplification, and (3) sequence less than 98% similarity in comparison with the transcriptome. The optimized SSRs were used to analyze the genetic diversity of 16 *P. violascens* varieties and 10 *Phyllostachys* species.

### Genetic Diversity of *P. violascens*

We used 54 primer pairs for the 16 *P. violascens* varieties, generating 399 alleles, which were treated as informative loci for dendrogram construction (Appendix 4). The number of SSR alleles was 3 to 12 for one primer pair, and each primer pair had 6.88 alleles, on average.

**FIGURE 1:**
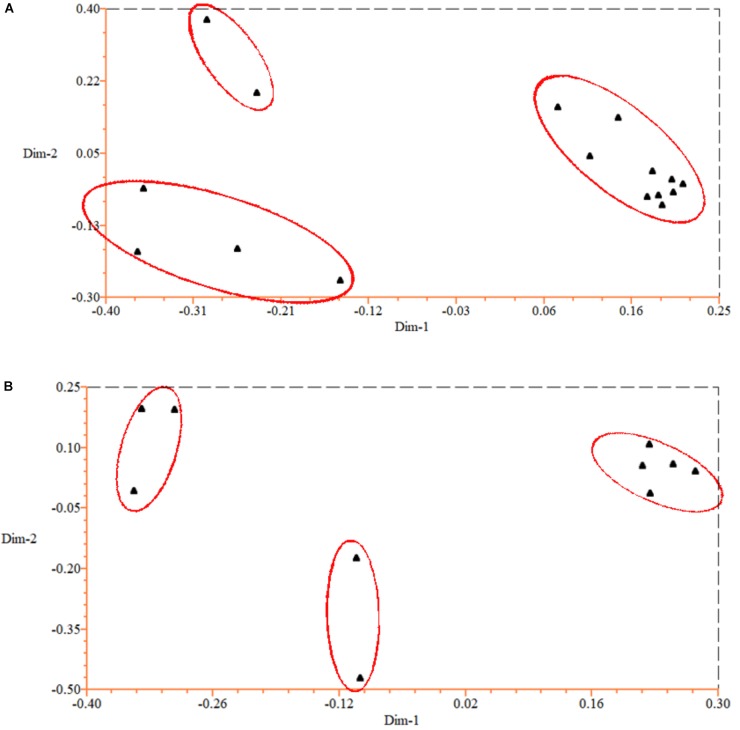
Principal coordinates analysis (PCoA) of *Phyllostachys violascens* based on EST-SSR data for **(A)** 16 *P. violascens* varieties and **(B)** 10 *Phyllostachys* species.

**FIGURE 2:**
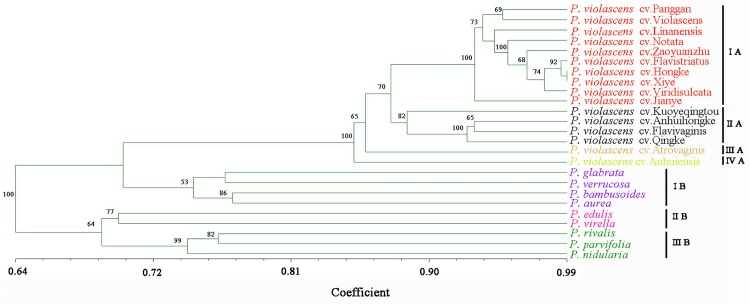
The dendrogram of bamboo species/varieties based on EST-SSR markers of *P. violascens*. Genetic relationship among **(A)** 16 varieties of *P. violascens* and **(B)** 10 species of *Phyllostachys*. The Roman numerals on the right side of the dendrogram represented the clustered groups. Bootstrap values over 50 were labeled on the branches of the dendrogram.

To test the genetic similarity between varieties, we used PCoA based on SSR data and found three groups among the 16 *P. violascens* varieties (Figure 1A). The phylogenetic tree was further constructed by using NTSYSpc (Figure 2A); the resulting Jaccard similarity coefficient ranged from 0.73 to 0.98. Four groups were uncovered in previous research according to genetic diversity of *P. violascens* varieties by combining ISSR, SRAP and AFLP marker analysis (Lin et al., 2011). For better comparison with previous study, the index 0.78 was selected and four main groups were detected in present study: groups I, II, III, and IV. Group I contained 10 varieties. Among the 10 varieties, *P. violascens* cv. Jianye and cv. Linanensis exhibited far genetic distances from other eight varieties; *P. violascens* cv. Hongke and cv. Xiye had very close genetic distance, with similarity index about 0.98. Among the four varieties in group II, the greater distant genetic distance was between *P. violascens* cv. Kuoyeqingtou and three other varieties. The groups III and IV contained only one variety each. These results were largely consistent with the PCoA results, with groups III and IV in the dendrogram clustered into one group on PCoA.

### Genetic Diversity of *Phyllostachys* Bamboo

The above 54 primer pairs were used with the 10 *Phyllostachys* bamboo species to verify their cross-amplification potential in *Phyllostachys*. The SSR primer pairs of *P. violascens* were perfectly applied in other *Phyllostachys* species. The number of SSR alleles ranged from 3 to 20, with an average of 11.3 per primer pairs. A total of 656 loci were used to construct the dendrogram of *Phyllostachys*; no missing data were found in the study (Appendix 5). As shown in the phylogenetic tree (Figure 2B), the Jaccard similarity coefficient ranged from 0.67 to 0.84. In this study, the 10 *Phyllostachys* species were separated into three distinct groups: I, II, and III. Group I contained five species: *P. violascens*, *P. glabrata*, *P. verrucosa*, *P. bambusoides*, and *P. aurea*. The Jaccard similarity coefficient was up to 0.84 between *P. violascens* and *P. glabrata*. Group II included two bamboo species: *P. edulis* and *P. virella*. Group III contained three bamboo species: *P. rivalis*, *P. parvifolia* and *P. nidularia*. Similar grouping results were shown by PCoA (Figure 1B).

## Discussion

### Characterization of EST-SSRs in *P. violascens*

Simple sequence repeat markers have been widely used in fingerprint construction, genetic diversity analysis and molecular marker-assisted breeding because of their high polymorphism, repeatability and codominant inheritance (Ran et al., 2010; Feng et al., 2018; Shi et al., 2018; Wang et al., 2018). However, traditional methods for developing SSR markers are not very efficient (Tang et al., 2010). Next-generation sequencing technologies can generate massive data, which are good resources for developing SSR markers in many species including bamboo (Tang et al., 2010; Abhishek et al., 2016; Zhang et al., 2017). Nevertheless, SSRs in *P. violascen* have not been reported because of lack of transcriptome or genome sequences. In this study, we identified 18,356 EST-SSR loci from 15,600 unigenes, and a total of 11,264 primer pairs were designed based on the transcriptome of *P. violascens.* Overall, 54 primer pairs were successfully used for identifying 16 *P. violascens* varieties and 10 *Phyllostachys* species, so the transcriptome sequences were good resources for developing SSR markers.

The frequency of SSRs in *P. violascens* was 1/4.55 kB when including the mononucleotide repeats, which was close to wheat (1/5.46 kB) (Peng and Lapitan, 2005) but significantly higher than in *Arabidopsis* (1/13.83 kB) (Cardle et al., 2000). Various factors may affect SSR frequency, including search criteria, software, as well as species properties (Sorrells, 2005). Tectonic activities and climate fluctuations during species evolutionary history contributed to the production and accumulation of genetic variations. Multi-locus plastid phylogenic analyses of bamboo revealed that the Bambusoideae began to diversify at about 43.26 million years ago (Mya), followed by the rapid radiation of the Arundinarieae at about 12–14 Mya, which was supposed to be induced by the important strengthening of the East Asian monsoon in the late Miocene (Zhang et al., 2016). Therefore, the high frequency of SSRs detected in *P. violascens* may be due in part to the long and complex evolutionary process of bamboo.

The types of repeat motifs in this study were not uniformly distributed in the *P. violascens* transcriptome database (Table 1). Di- and tri-repeats were the most predominant when excluding mononucleotide repeats (Table 2). This result differed from Ma bamboo (*Dendrocalamus latiflorus*), in which tri-repeats are the most abundant (Abhishek et al., 2016). In addition, the proportion of tetra-, penta- and hexanucleotide repeats was significantly lower in *P. violascens* than *D. latiflorus* (Table 2). The differentiation between *P. violascens* and *D. latiflorus* was likely caused by distant genetic distance. *Phyllostachys* belong to the Arundinarieae, a kind of temperate woody bamboo, and *Dendrocalamus* is affiliated with tropical woody bamboo Bambuseae (Soreng et al., 2014). These two tribes diversified at the beginning of the bamboo evolutionary history and preserved distinct genetic elements (Zhang et al., 2012, 2016).

**Table 2:** Comparison of frequency of microsatellites of *Phyllostachys violascens*, *Dendrocalamus latiflorus, Arabidopsis thaliana*, and *Triticum aestivum.*

Plant	*P. violascens*	*D. latiflorus*	*A. thaliana*	*T. aestivum*
Total	9,257	22,305	1,070	43,598
Di-	48.06%	16.10%	26.27%	20.77%
Tri-	48.84%	47.70%	73.04%	74.26%
Tetra-	2.54%	26.10%	0.72%	3.36%
Penta-	0.42%	6.90%	0	1.12%
Hexa-	0.14%	3.30%	0	0.50%


As shown in Table 1, the AG/CT motif was the most predominant di-repeat (17.11%), which was similar to pigeonpea (16.7%) (Dutta et al., 2011) but higher than wheat (8.7%) (Peng and Lapitan, 2005) and lower than taro (52.86%) (You et al., 2015). Among tri-repeats, CCG/CGG was the most abundant (5.91%), which was consistent with previous findings in taro (You et al., 2015), rice and maize (Cardle et al., 2000). The AGC/GCT type (2.94%) was the second most abundant tri-repeat, which was not common in taro (You et al., 2015), rice or maize (Cardle et al., 2000). Previous studies showed that the tri-repeat type of CCG/CGG was a rare motif in dicotyledonous plants but the most abundant repeat type among the tri-repeats in monocots (Varshney et al., 2002; You et al., 2015). Our results verified that many CCG/CGG repeats was a common feature of monocot plants and the high G/C content plays an important role in monocots (Morgante et al., 2002; Rota et al., 2005; Wang et al., 2010).

### Genetic Diversity in 16 *P. violascens* Varieties

The genetic diversity of *P. violascens* germplasm has been unclear. The classification of 16 varieties of *P. violascens* previously involved using three distinct types of molecular markers - ISSR, SRAP and AFLP individually – but failed (Lin et al., 2011). When combining ISSR, SRAP and AFLP, these 16 varieties could be identified successfully (Lin et al., 2011). In the present study, the 16 varieties of *P. violascens* could also be distinguished by polymorphic EST-SSR markers, and significant genetic variations between different *P. violascens* varieties were uncovered. These results implied that SSR markers and the combination of ISSR, SRAP and AFLP markers were more sensitive than using the ISSR, SRAP and AFLP markers alone. Considering the convenience of experimental design, the SSR marker method is more applicable than ISSR, SRAP and AFLP combined.

In addition, both PCoA and phylogenetic analysis revealed similar grouping results for the 16 *P. violascens* varieties based on SSR markers (Figures 1A, 2A). The cluster results for SSR markers were similar to combining ISSR, SRAP and AFLP markers but differed little when using ISSR, SRAP, and AFLP markers alone (Lin et al., 2011). Hence, the SSR method and combining ISSR, SRAP and AFLP markers were more reliable than using ISSR, SRAP and AFLP markers alone. Meanwhile, the cluster result of SSR was more similar with AFLP than with ISSR and SRAP markers, which suggests that the AFLP method was more reliable than ISSR and SRAP methods in classifying *P. violascens*.

### Transferability of EST-SSR Markers and Genetic Diversity Among 10 *Phyllostachys* Species

To test the versatility of EST-SSR markers derived from *P. violascens*, the 54 SSR primer pairs were further used to analyze the genetic diversity of 10 *Phyllostachys* species. Clear bands were generated and higher transfer rate (100%) was detected by using these SSR primer pairs as compared with *Elymus sibiricus* (22.40%) (Zhou et al., 2016), *Chrysanthemum nankingense* (20%) (Wang et al., 2013), *Juglans mandshurica* (30.8%) (Hu et al., 2016), and *Neolitsea sericea* (16.3%) (Chen et al., 2015), which implies the availability of SSR markers in the genus of *Phyllostachys*. Furthermore, a phylogenetic tree encompassing all 16 *P. violascens* varieties and 9 other *Phyllostachys* species used in this study was also built based on the EST-SSRs markers (Appendix 6). The results showed that all varieties of *P. violascens* were clustered into one main group and kept separate with the other *Phyllostachys* species. The internal branch structure of the common dendrogram was consistent with the separate phylogenetic analysis based on the 16 *P. violascens* varieties (Figure 2A) and 10 *Phyllostachys* species (Figure 2B), confirming the versatility and robustness of the EST-SSR markers in distinguishing different varieties of *P. violascens* and different *Phyllostachys* species. Therefore, these EST-SSR markers might be used for classifying different species in *Phyllostachys* and even different genera of bamboo.

Figure 2B shows that these 54 SSR primer pairs developed from *P. violascens* could successfully distinguish the 10 *Phyllostachys* species. *Phyllostachys* has been divided into Sect. *Phyllostachys* and Sect. *Heterocladae* according to the *Flora of China* (Wu and Raven, 2006). In this study, 10 species of *Phyllostachys* were divided into three groups by using SSR markers of *P. violascens*. Groups I and II belonged to Sect. *Phyllostachys* and group III belonged to Sect. *Heterocladae*. However, species from groups II and III were clustered together. One reasonable explanation was that Sect. *Phyllostachys* is not monophyletic. Alternatively, given the lack of informative characters, the existence of incomplete lineage sorting, and/or interspecies hybridization, it is not unexpected that bamboo species from different sections clustered together based on molecular markers and genome data (Zhang et al., 2016). As well, the classification of Sect. *Phyllostachys* and Sect. *Heterocladae* in the *Flora of China* mainly depended on morphological characteristics, such as whether the blade was horizontal, reflexed or erect and whether culm sheaths were covered with spots. Further studies of the classification of Sect. *Phyllostachys* and Sect. *Heterocladae* based on both morphological characteristics and molecular analysis are needed.

## Author Contributions

KC and XL organized the research and wrote the experimental subjects. LZ and KZ participated in the research. LL and ZZ collected the sample and material. XL and WZ provided technical guidance and paper modification.

## Conflict of Interest Statement

The authors declare that the research was conducted in the absence of any commercial or financial relationships that could be construed as a potential conflict of interest. The reviewers FB and FG, and handling Editor declared their shared affiliation.

## Footnotes

1 http://pgrc.ipk-gatersleben.de/misa/misa.html

2 https://sourceforge.net/projects/primer3/

3 http://pgrc.ipk-gatersleben.de/misa/primer3.html

## Supplementary Material

APPENDIX 1 Frequency distribution of the EST-SSR repeat motifs in *P. violascens*.

APPENDIX 2 The all SSR primer pairs of *P. violascens*.

APPENDIX 3 The 54 primer pairs of *P. violascens* were applied in study.

APPENDIX 4 The two-dimensional matrix of *P. violascens* varieties informative loci.

APPENDIX 5 The two-dimensional matrix of *Phyllostachys* species informative loci.

APPENDIX 6 The dendrogram of *Phyllostachys* species and *P. violascens* varieties based on EST-SSR markers of *P. violascens*.
