# Supplementary figures and images for: Development and Characterization of EST-SSR Markers From RNA-Seq Data in Phyllostachys violascens
Source: Front Plant Sci. 2019 Feb 1;10:50. doi: 10.3389/fpls.2019.00050 (PMC6367221; doi:10.3389/fpls.2019.00050)

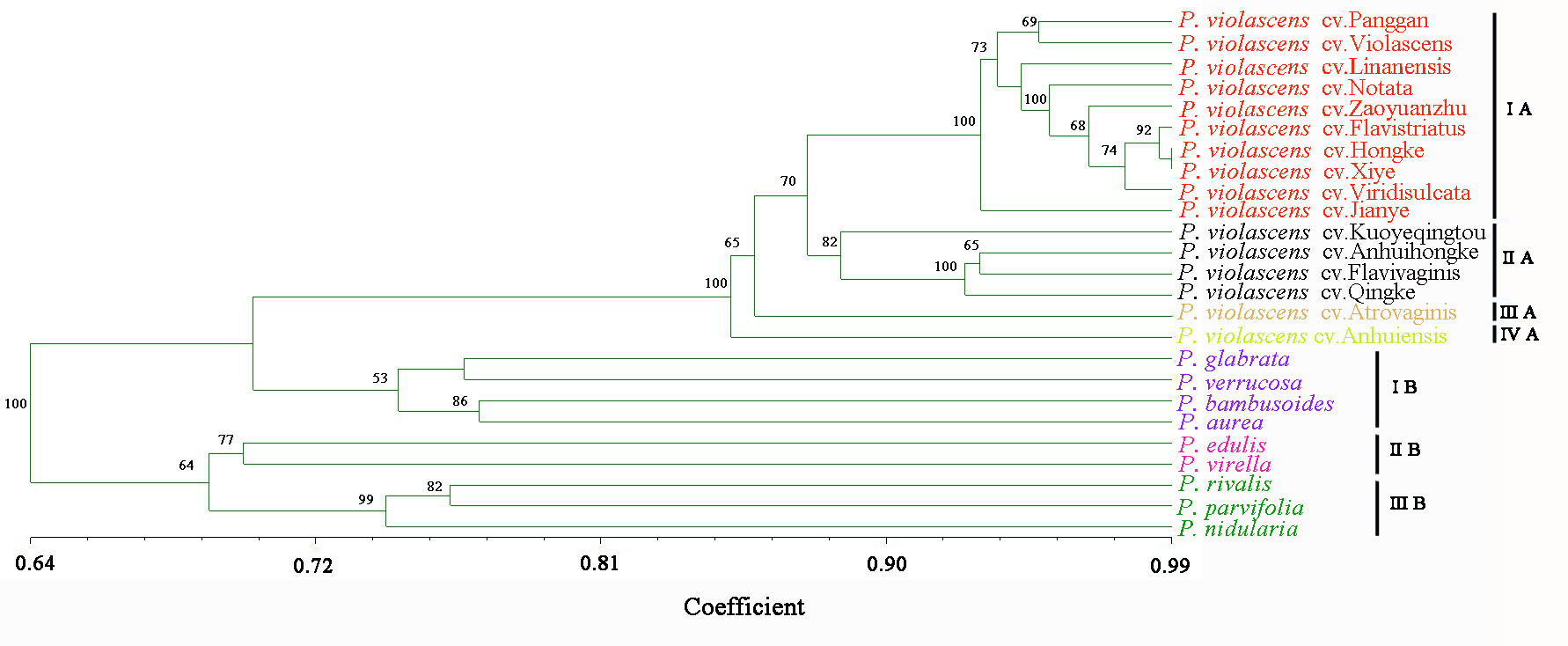

Supplement: APPENDIX 6 — The dendrogram of Phyllostachys species and P. violascens varieties based on EST-SSR markers of P. violascens. [file Data_Sheet_6.zip › Image 1.tif]
